# Number-Theoretic Methods in Statistics: Theory and Applications

**DOI:** 10.3390/e28020147

**Published:** 2026-01-28

**Authors:** Kai-Tai Fang, Yongdao Zhou

**Affiliations:** 1 Guangdong Provincial Key Laboratory of Interdisciplinary Research and Application for Data Science (IRADS), Beijing Normal-Hong Kong Baptist University, Zhuhai 519087, China; ktfang@bnbu.edu.cn; 2 The Key Laboratory of Random Complex Structures and Data Analysis, The Chinese Academy of Sciences, Beijing 100045, China; 3 School of Statistics and Data Science, Nankai University, Tianjin 300071, China

There are many settings in which the key underlying quantities that affect the outcome are unknown, resulting in the unknown inputs often being modeled using random vector variables or stochastic processes. The essence of the Monte Carlo (MC) method is to estimate the mean, quantiles, and/or probability distribution of the outcome. An enhancement of the traditional MC method, the Quasi-Monte Carlo (QMC) method, is a numerical technique used to approximate integrals, particularly in high-dimensional spaces [1,2]. Instead of using the random points of the MC method, QMC methods employ low-discrepancy sequences (also called quasi-random sequences or sub-random sequences), such as the Halton sequence, the Sobol sequence, or the Faure sequence, to achieve faster convergence. Quasi-Monte Carlo method has a rate of convergence close to O(1/N), whereas the rate for the traditional Monte Carlo method is $O(N^{-0.5}$). QMC methods are also called number-theoretic methods (NTMs), and have played an important role in numerical integration in high dimensions, statistical inference, and experimental design, as well as having applications in engineering, biology, economics, and data science.

NTMs are a class of methods that represent a combination of number theory and numerical analysis [3]. Since the 1980s, statisticians have directed their attention to NTMs and their applications in statistics, with the first application of NTMs being an evaluation of the probabilities and moments of a multivariate distribution. Later, Shaw [4] provided a detailed discussion on applications of NTMs in Bayesian statistics, mainly for the numerical computation of posterior density and moments. Fang [5] was the first to apply NTMs to the design of experiments, and proposed new type of design called uniform design. With the exception of integration, this was the first application of NTMs in statistics. Since then, the theory and applications of uniform design developed quickly in China and in other countries. Several monographs on the topic of uniform design were published, see [6,7,8]. The content of uniform design has also been included in many encyclopedia or handbooks, such as the *Handbook on Statistics*, the *Handbook of Engineering Statistics*, and the *International Encyclopedia of Statistical Science*. Uniform experimental design can be understood as a space-filling design for computer experiments, a fractional factorial design with model uncertainty, a robust design against model specification, and a supersaturated design, and an experimental design that can be applied to experiments with mixtures.

The essence of NTMs is to find representative points (RPs) of the uniform distribution in the experimental domain, such as the unit cube of $R^{s}$, the simplex, or another given region. In other words, the main idea of representative points in NTMs is to scatter the point set uniformly in the experimental domain. Those RPs are also called space-filling designs in the field of experimental design [8,9]. Many criteria, such as the uniformity criteria [10,11], maximin distance criterion [12], and low-dimensional projection uniformity [13], have been proposed to assess the space-filling properties of design points. Recently, NTMs have been extended to generate RPs for many useful multivariate distributions and have been systematically applied in statistics [14]. Therefore, the lively interest in the development of the theory and application of NTMs has prompted us to propose this Special Issue on “Number-Theoretic Methods in Statistics: Theory and Applications”, which brings together ten contributions that address the topic of NTMs from different methodological and applicative perspectives. Among them, four contributions cover the space-filling properties of factorial designs, as well as some other factorial design-related criteria; four contributions center on the RPs for different distributions; and two contributions focus on the application of NTMs.

As shown in [15], the uniform criterion has a close relationship with generalized minimum aberration criterion of fractional factorial designs. Therefore, discussions of the criteria of factorial design are useful in terms of their space-filling properties, the paper on the “Construction of Space-Filling Asymmetrical Marginally Coupled Designs” by Zhou et al. (Contribution 1) studies marginally coupled designs (MCDs) for computer experiments with both qualitative and quantitative factors, and proposes four approaches to constructing a series of space-filling asymmetrical MCDs based on space-filling symmetrical MCDs or space-filling Latin hypercube designs (LHDs). The obtained asymmetrical MCDs can inherit the low-dimensional space-filling properties of these symmetrical MCDs or LHDs, and are flexible in terms of their run sizes.

In “Maxpro Designs for Experiments with Multiple Types of Branching and Nested Factors”, Yang and Zhou (Contribution 2) considered the space-filling design of experiments with branching factors and nested factors by using the maxpro criterion, which is also a type of space-filling criterion. They proposed this novel space-filling criterion based on the maximum projection criterion to evaluate the performance of designs with branching and nested factors, and provided a framework to construct optimal designs under the proposed criterion. Compared with the existing works, the resulting designs have superior space-filling properties across all possible low-dimensional projections. Moreover, their strategy is flexible regarding run size, number of levels, and type of factors, ultimately improving the space-filling properties of such designs.

The paper on “Two-Level Regular Designs for Baseline Parameterization” by Zhao and Qin (Contribution 3) considers the baseline parameterization model of two-level regular fractional factorial designs. The baseline parameterization model is a linear model based on baseline constraints, and is one of the most used models in the analysis of experimental data. The authors made progress on bridging the *K*-values and word length pattern, and analytically calculate the quantities for two-level regular designs with a higher resolution, which will result in enhanced space-filling properties.

Moreover, a new three-level aliasing pattern was proposed in the paper “An Aliasing Measure of Factor Effects in Three-Level Regular Designs” by Chen, Li and Li (Contribution 4). The aliasing properties of factor effects are more significant than the component effects in the experimental model. The proposed new aliasing pattern for three-level regular designs can be used to evaluate the degree of aliasing among different factors, as well as choosing optimal three-level regular designs. The proposed criterion has a strong relationship with other commonly used criteria such as general minimum lower-order confounding, entropy, minimum aberration, and clear effects. Moreover, the optimal three-level design for all 27-run and some 81-run and 243-run three-level designs is listed in tables, and can be used for practical applications.

Traditional NTMs consider the point set as the representative points of uniform distribution in experimental domains. Currently, the theory of representative points has been extended to other useful distributions, such as the normal distribution, exponential distribution family, skew-*t* distribution, and so forth. To search the representative points of a given distribution, several criteria have been proposed. The simplest method is to use the Monte Carlo method, the corresponding RPs are then called MC-RPs. Fang and Wang [6] were the first to explore statistical simulation using NTMs. They constructed approximate distributions to F(x) via QMC-based representative points (QMC-RPs). They also considered the representative points under the mean square error (MSE) criterion, the corresponding RPs are called MSE-RPs or principal points [16]. The properties of MC-RPs, QMC-RPs, and MSE-RPs, and their application in statistical simulation, experimental design, geometric probability problems, and finance, have been studied in [14]. Statistical simulation has become a cornerstone in statistical research and applications, and it has been shown that the converge rate can be improved when replacing MC-RPs with QMC-RPs or MSE-RPs. This Special Issue includes four contributions on RPs.

In the paper “The Representative Points of Generalized Alpha Skew-*t* Distribution and Applications” by Zhou et al. (Contribution 5), the authors studied the construction methods and properties of different types of RPs of the generalized alpha skew-*t* distribution, such as MC-RPs, QMC-RPs, and MSE-RPs. These three types of RPs are utilized to estimate moments and densities of special distributions with known and unknown parameters, and the authors were able to show that the MSE-RPs perform best across all case studies.

MSE-RPs were also discussed in the paper “Mean Squared Error Representative Points of Pareto Distributions and Their Estimation” by Li and Peng (Contribution 6). Pareto distributions are widely applied in various fields, such as economics, finance, and environmental studies, and this paper demonstrates the uniqueness and existence of MSE-RPs in Pareto distributions under certain parameter settings, as well as providing a theoretical *k*-means algorithm for the computation of MSE-RPs for Pareto I and Pareto II distributions. Several construction methods for estimating MSE-RPs in different types of Pareto distributions are provided. The effectiveness of the proposed methods for MSE-RPs is compared through simulations and real data studies.

The study “Advancing Continuous Distribution Generation: An Exponentiated Odds Ratio Generator Approach” by Chen et al. (Contribution 7) presents a new methodology for generating continuous statistical distributions, integrating the exponentiated odds ratio within the framework of survival analysis. The benefit of the new method is illustrated through the mathematical properties and statistical properties of the Type 2 Gumbel Weibull-*G* family of distributions, such as density functions, moments, hazard rate and quantile functions, Rényi entropy, order statistics, and the concept of stochastic ordering.

In the paper “A Review: Construction of Statistical Distributions”, Fang, Lin, and Deng (Contribution 8) focused on the methods used to construct discrete or continuous and univariate or multivariate distributions, covering both traditional and newly developed approaches. They consider the classic distributions such as the normal, exponential, gamma, and beta for univariate data, and the multivariate normal, elliptical, and Dirichlet for multidimensional data, and showed these to be necessary for more flexible modeling tools, as well as their construction methods. The approximation to the continuous distributions by different types of representative points is also discussed.

Two contributions to this Special Issue concern the applications of NTMs. The study “Maximum Entropy-Minimum Residual Model: An Optimum Solution to Comprehensive Evaluation and Multiple Attribute Decision Making” by Tang and Lin (Contribution 9) considers the weighting methods of the factors. The authors propose a new model to generate weights based on the maximum entropy–minimum residual (MEMR) principle, directly estimating the relationship between factor weights and the composite indicator. Compared with other weighting methods, the MEMR model is more robust, consistent, and interpretable, and is therefore suitable for all comprehensive evaluation cases involving quantitative factors.

Furthermore, in the paper “Federated Semi-Supervised Learning with Uniform Random and Lattice-Based Client Sampling”, Zhang and Yang (Contribution 10) propose a novel federated averaging semi-supervised learning algorithm, called FedAvg-SSL, by using two sampling approaches that rely on MC-RPs and QMC-RPs. The lattice-based sampling method using QMC-RPs proved to provide more balanced client participation through structured deterministic selection. The rigorous convergence analysis showed that FedAvg-SSL achieves a sublinear convergence rate with linear speedup, which was validated through simulation experiments.

Overall, this Special Issue presents a variety of methodologies and applications of number-theoretic methods in statistics, including the construction methods of space-filling designs, different types of representative points such as QMC-RPs and MSE-RPs, and their applications in modeling techniques and federated semi-supervised learning. The benefits of NTMs were valuated according to different aspects in the collected contributions. Further properties, construction methods, and applications of NTMs are welcome in future editions of this Special Issue.
